# Structure matters: Assessing the statistical significance of network topologies

**DOI:** 10.1371/journal.pone.0309005

**Published:** 2024-10-02

**Authors:** Bernat Salbanya, Carlos Carrasco-Farré, Jordi Nin

**Affiliations:** 1 Universitat Ramon Llull, Esade, Avinguda de la Torre Blanca, Catalonia, Spain; 2 Information Systems Department, Toulouse Business School, Toulouse, Occitanie, France; University of Geneva: Universite de Geneve, SWITZERLAND

## Abstract

Network analysis has found widespread utility in many research areas. However, assessing the statistical significance of observed relationships within networks remains a complex challenge. Traditional node permutation tests are often insufficient in capturing the effect of changing network topology by creating reliable null distributions. We propose two randomization alternatives to address this gap: random rewiring and controlled rewiring. These methods incorporate changes in the network topology through edge swaps. However, controlled rewiring allows for more nuanced alterations of the original network than random rewiring. In this sense, this paper introduces a novel evaluation tool, the Expanded Quadratic Assignment Procedure (EQAP), designed to calculate a specific p-value and interpret statistical tests with enhanced precision. The combination of EQAP and controlled rewiring provides a robust network comparison and statistical analysis framework. The methodology is exemplified through two real-world examples: the analysis of an organizational network structure, illustrated by the Enron-Email dataset, and a social network case, represented by the UK Faculty friendship network. The utility of these statistical tests is underscored by their capacity to safeguard researchers against Type I errors when exploring network metrics dependent on intricate topologies.

## Introduction

Understanding network processes is crucial for uncovering the emergent behavior of interconnected elements [1]. Usually, examining individual actions provides insights, but it falls short of grasping broader global dynamics [2]. Network science aids researchers in understanding complex interactions among elements [3]. However, studying networks raises critical questions, particularly regarding statistical limitations and the role of network topology when interpreting observed outcomes [4]. This acknowledgment underscores the need for a more gradual approach when analyzing if there are significant differences between diverse network topologies.

In this sense, hypothesis testing in network analysis represents a growing area where statistical methods intersect with complex data structures. Exploring different methodologies has been a subject of significant interest and has garnered attention within the research community. While some contributions have been instrumental in extending standard testing frameworks to network-based hypothesis assessments [5, 6], or adapted resampling techniques [7] to network-based data, others have paved the way for incorporating Bayesian frameworks in complex networks’ hypothesis assessments [8, 9]. However, further developments are still required.

Irrespective of the hypothesis testing framework employed, permutation tests have consistently served as a primary tool for generating alternative networks. These alternative networks play a crucial role in evaluating the significance of the topological properties in the studied network, including centrality [10], interconnectivity [11, 12], subgraph patterns [13, 14], and network dynamics [15, 16]. By facilitating an exploration of the link between network structure and model outcomes, they greatly enhance result interpretation and enable more accurate predictions about the behavior of the system under analysis [10]. Such capability arises from permutation tests, which randomly shuffle the network structure while holding other variables constant, generating a null distribution for comparative analysis. [17, 18]. At last, researchers can determine the statistical significance of the network topology by comparing observed network metrics with those generated through permutations.

However, permutation tests may not always provide accurate results since not all changes have the same impact, as demonstrated in the limitations section. Misinterpreting Type I errors in network analysis can lead to false positive findings, resulting in erroneous conclusions, spurious associations, and misleading downstream analyses. Therefore, selecting appropriate statistical methods is crucial to ensure the integrity of the obtained results [19].

A well-known combinatorial optimization problem that has garnered significant attention due to its challenging nature and diverse real-world applications is the *Quadratic Assignment Procedure* (QAP). The QAP is a method primarily used for testing dyadic hypotheses. It is essential to note that QAP is not designed to test structural elements like motifs, centralities, or clustering within a network. The nature of the hypotheses that QAP can address is crucial for understanding its applicability. QAP, being a permutation-based method, is commonly employed for conducting dyadic regression. This method is particularly interesting for analyzing relationships between pairs of entities in various contexts [20]. In this sense, some recent studies suggest that combining edge rewiring with the existing techniques could be beneficial for testing significant differences between the original network and the rewired version [21]. By incorporating edge rewiring, researchers can introduce controlled variations into the network structure, allowing for comparing the original network with modified versions. This approach enables the assessment of how changes in edge connections impact the network’s overall characteristics and properties.

In light of this limitation, this article presents a novel approach to evaluating the statistical significance of a given network topology. Our contributions extend to three main areas:

1. We introduce a new method, the Expanded Quadratic Assignment Procedure (EQAP), to quantify network similarity more effectively than traditional methods such as the Mantel test and the Quadratic Assignment Procedure (QAP). These conventional methods fall short in analyzing the complex topology of networks.
2. To address this, we explore two randomization alternatives. First, the well-known “*random rewiring*”, which retains the number of connections for each network element while randomly reconnecting them. Second, we introduce an alternative technique called “*controlled rewiring*”, which involves reconnecting elements, starting with those with fewer central connections and progressively including more central ones.
3. The primary focus of our research is to analyze the p-values obtained through combining these alternatives with the EQAP method. Traditional permutation methods result in overly significant p-values, whereas our proposed alternatives maintain a balance, enabling more accurate comparisons.
4. By integrating Random Rewiring and Controlled Rewiring into the EQAP, we offer a comprehensive toolset for network analysis. The primary objective is to generate multiple non-significant randomized versions of the original network for subsequent comparative modeling.
5. Our results indicate that controlled rewiring is the most suitable method for analyzing the significance of topological metrics in networks. By systematically reconnecting nodes based on their connectivity, this method enables a detailed examination of how network topology impacts network dynamics.
6. We have developed a user-friendly Python library to facilitate the assessment of network topology significance by allowing researchers to compare the original network metrics with those derived from the rewired networks.

The rest of this paper is organized as follows. Firstly, we introduce the main topological metrics used in network research and revise the existing methods to analyze network topological significance. Secondly, we examine their limitations when comparing a modified network to the original one. Then, we propose two randomization alternatives to alter the network’s topology, and the Expanded Quadratic Assignment Procedure (EQAP), which measures whether there are significant differences with a p-value. Subsequently, the process of implementing the proposed methodology, guide on comparing it against a Null Model, and derived experimental results on real-world data. Finally, we discuss the methodological findings, recommendations when implementing the methods, and some research conclusions and future work.

## Related work

Networks can exhibit diverse structural configurations. In this work, we consider a network as a pair *G* = (*V*, *E*), where *V* is a set of *nodes* or vertices connected by a set of *E* *edges* or links [22, 46]. The *adjacency matrix* of a network *G* is a square matrix representing the connections or relationships between the nodes in the graph. Let *n* be the number of vertices. If the vertices are labeled *V* = *v*_1_, *v*_2_, …, *v*_*n*_, then the adjacency matrix *A* will be an *n* × *n* matrix. The elements *a*_*ij*_ ∈ *A* equal 1 if an edge exists between vertex *v*_*i*_ and vertex *v*_*j*_, or 0 otherwise.

The number of nodes, edges, or the network type (directed or undirected) are measures of network complexity. However, other metrics, such as the *degree* distribution, shed more light on its connectivity and functionality. The **degree** ($k_{v_{i}}$) corresponds to the number of edges a node *v*_*i*_ has to other nodes *v*_*j*_ [23]. In the case of directed networks, we can calculate each node’s inward degree, or in-degree, ($k_{v_{i}}^{in}$) and outward degree, or out-degree, ($k_{v_{i}}^{out}$).

Several metrics are used to study network topology features. Centrality measures describe how predominant nodes are located in the center of any network. The most common centrality measure is **betweenness**. It is calculated as $B_{v_{i}}=\sum_{v_{j}\neq v_{i}\neq v_{k}}\frac{\sigma_{\left(v_{j},v_{k}\right)}\left(v_{i}\right)}{\sigma_{\left(v_{j},v_{k}\right)}},$ where $\sigma_{\left(v_{j},v_{k}\right)}$ stands for the total number of shortest paths from node *v*_*j*_ to node *v*_*k*_ and $\sigma_{\left(v_{j},v_{k}\right)}(v_{i})$ is the number of those paths that pass through *v*_*i*_. Betweenness centrality can also be calculated on an edge’s basis, which we call **edge betweenness**. In addition, the **closeness** metric is a simplified version of betweenness. It measures how central a node is by considering the total distance between a given node and all the others. Closeness was defined by [24] as $C_{v_{i}}=\frac{1}{\sum_{i}d\left(v_{j},v_{i}\right)},$ where *d*(*v*_*j*_, *v*_*i*_) stands for the length of the shortest path between nodes *v*_*i*_ and *v*_*j*_.

Additionally, to centrality measures, interconnectivity measures provide valuable insights into resource flow efficiency. The **average shortest path length** assesses the network’s global connectivity and diameter by quantifying the average distance between every pair of nodes. The **local clustering coefficient** [25] indicates the level of interconnectivity among a node’s neighbors, and it represents the probability that two neighbor nodes connect each other [23]. For a specific node *v*_*i*_ with degree $k_{v_{i}}$, it is defined as $C_{v_{i}}=\frac{2L_{v_{i}}}{k_{v_{i}}\left(k_{v_{i}}-1\right)},$ where $L_{v_{i}}$ stands for the number of edges among *v*_*i*_ neighbors. The average value of local clustering coefficients is often called *transitivity* [23].

Besides, **Assortativity** refers to the tendency of nodes in a network to connect to similar nodes. To measure assortativity, Newman (2002) developed the **assortativity coefficient** [26]. When considering the node degree in directed networks, it is calculated as $r=\frac{\sum_{v_{i}}e_{\left(v_{i},v_{i}\right)}-\sum_{v_{i}}k_{v_{i}}^{in}k_{v_{i}}^{out}}{1-\sum_{v_{i}}k_{v_{i}}^{in}k_{v_{i}}^{out}},$ where $\sigma_{\left(v_{i},v_{i}\right)}$ is the self-loops sum of the node *v*_*i*_ and $k_{v_{i}}^{in}$, and $k_{v_{i}}^{out}$ are the fraction of incoming and outgoing edges attached to the node *v*_*i*_.

Finally, other measures study how subgraph patterns arise within a network topology. The **number of triangles** in a network measures the local interconnectivity density and represents mutual connections between nodes [23]. Triangles are indicative of cohesive social groups or subnetworks. It is computed as the number of sets of three nodes, each with a relationship to the other two. The **global clustering coefficient** quantifies the proportion of closed triangles about the total number of triangles (both open and closed) in a network.

Some studies also analyze network similarities by focusing on the existence of common structural patterns or motifs shared across networks belonging to the same superfamily [27]. These similarities, known as Structural Patterns (SPs) of superfamilies, suggest underlying commonalities in the local structure or connectivity patterns among networks, even when describing different systems. Identifying such SPs provides insights into potential functional tasks or evolutionary relationships among networks within the same superfamily. Under this perspective, our approach offers an alternative methodology for investigating network dynamics and properties, i.e., instead of focusing on identifying similarities in SPs, we propose integrating statistical analysis with systematic alterations of network topology by comparing the similarities between networks before and after structural alterations induced by edge rewiring. This comparison attempts to shed light on how changes in network topology influence network behavior, robustness, and function.

To assess the meaning of the presented metrics and their implications, subjecting them to a statistical test against a null distribution is essential. To this end, hypothesis testing methodologies have been extensively discussed [28–30], which provided the theoretical foundations and practical applications of statistical hypothesis testing, offering insights into optimal testing procedures and efficiency.

Nowadays, the most commonly used tests for network analysis in social sciences are the Mantel test, presented here for completeness of the analysis, and the Quadratic Assignment Procedure (QAP) [31, 32]. These tests help researchers determine whether the observed patterns and relationships in network data are statistically significant or could have occurred by random chance (the null hypothesis). In essence, they serve as critical tools for verifying the validity and significance of network analyses [33].

### Mantel test

The Mantel test is a statistical method frequently employed in network analysis to assess the similarity or dissimilarity between two networks through permutations [31]. This test is commonly used to compare two different networks based on their structure and assess the significance of their differences [34]. Given the null hypothesis that there is no correlation between two distance matrices of the same size, the Mantel test rejects this hypothesis, as explained below.

$$\begin{array}{c} r_{M}=\frac{\sum_{i<j}w_{ij}d_{ij}}{\sqrt{\sum_{i<j}w_{ij}^{2}\sum_{i<j}d_{ij}^{2}}}, \end{array}$$ (1)

where the indices *ij* refer to the element in the *i*-th row and *j*-th column of the matrices. The numerator of *r*_*M*_ represents the sum of the products of the corresponding upper triangle elements of the matrices *W* and *D*, while the denominator represents the product of their standard deviations.

To test the null hypothesis, we generate a null distribution of *r*_*M*_ by permuting the rows and columns of *D* and recalculating *r*_*M*_. Specifically, we randomly permute the rows and columns of *D* for *B* times and calculate $r_{M}^{\left(b\right)}$ for each permutation *b* = 1, …, *B*. We then calculate the empirical p-value as:

$$\begin{array}{c} p=\frac{\sum_{b=1}^{B}I\left(r_{M}^{\left(b\right)}\geq r_{M}\right)+\sum_{b=1}^{B}I\left(r_{M}^{\left(b\right)}\leq -r_{M}\right)}{2B}, \end{array}$$ (2)

where *I*(⋅) is the indicator function. If *p* is below a pre-specified significance level *α*, the null hypothesis is rejected, concluding there is a significant correlation between *W* and *D*.

### Quadratic Assignment Procedure

An alternative method is the Quadratic Assignment Procedure (QAP). QAP is a permutation-based method used to test the significance of the association between two matrices in network analysis. It is useful for assessing the similarity or dissimilarity between two networks by comparing the observed matrix similarity/dissimilarity with a null distribution obtained through matrix permutations [35, 36]. QAP can handle various types of matrices, including adjacency and similarity matrices. When using adjacency matrices, QAP tests hypotheses about the relationship between different types of networks or the same network observed at different times or under different conditions. This flexibility makes QAP a powerful tool for network analysis.

To account for the dependency structure of network data, QAP generates a null distribution by randomly shuffling the rows and columns of the matrices while preserving the dependencies within each matrix. This approach creates a null distribution, assuming no association between the matrices. By using matrix permutations that respect the dependencies, QAP provides a robust method for testing the significance of the association, addressing the non-independence of network data [32, 34].

The Quadratic Assignment Procedure (QAP) runs as a combinatorial optimization method targeting a specific challenge. Given two *n* × *n* matrices, *W* = (*w*_*ij*_) and *D* = (*d*_*ij*_), the goal of the Quadratic Assignment Procedure is to find a permutation matrix *P* of size *n* × *n* that minimizes the objective function:

$$\begin{array}{c} \text{Obj}\left(W,D\right)=\text{trace}\left(W^{T}DP\right) \end{array}$$ (3)

where trace denotes the trace of a matrix, and *P* is a permutation matrix, i.e., a binary matrix with exactly one 1 in each row and each column and all other entries being 0. The (*i*, *j*)-th entry of *P* is denoted by *p*_*ij*_, which is 1 if and only if an element of column *i* is assigned to column *j*.

The QAP finds the optimal permutation matrix *P* that minimizes the objective function, effectively assigning rows and columns of matrix *W* to rows and columns of matrix *D* in a way that minimizes the objective of the assignment, as measured by the trace of the product *W*^*T*^*DP*.

The QAP for 2D matrices is based on the following assumptions:

- If *P* is a permutation matrix, then *P*^*T*^ is also a permutation matrix.
- The trace of a product of two matrices is equal to the sum of the products of their corresponding elements.
- The objective function can be written as $\text{Obj}\left(W,D\right)=\sum_{i=1}^{n}\sum_{j=1}^{n}w_{ij}d_{p_{i},p_{j}}$, where *p*_*i*_ and *p*_*j*_ are the indices of the elements in *D* that correspond to the assigned locations of *i* and *j* under the permutation matrix *P*.

## Limitations of the existing permutation tests

Permutation tests, including the Mantel test and QAP, are invalid for detecting minor differences in network topologies. These tests rely on randomly permuting the data to create a null distribution. However, when the differences between network topologies are subtle, the procedure fails to effectively capture these small changes, leading to limited statistical power and inaccurate inference [37].

### Limitations of the Mantel test

The Mantel test encounters an issue when comparing permutations of the same matrix. In such cases, the test rearranges the order of pairwise distances without altering the actual distances between pairs of observations. While traditionally associated with distance matrices, the Mantel test is versatile and applicable to any type of square symmetric matrix, including adjacency matrices. Consequently, the correlation between the matrices becomes perfect, and the resulting p-value equals one, indicating no evidence of a significant difference between the matrices [32, 34].

#### Limitation 1

**The Mantel test p-value for equal matrices** (*D* = *W*) **is always 1**. Consider two distance matrices, *W* and *D*, both sized *n* × *n*. The Mantel test statistic, denoted as *r*_*M*_, is defined by the covariance between *W* and *D*, divided by the product of their standard deviations.

Expressed as $r_{M}=\frac{\text{cov}(W,D)}{\sigma (W)\cdot \sigma (D)}$, where cov and *σ* represent covariance and standard deviation functions, respectively. When *W* equals *D*, the covariance simplifies to the variance of *W*. Substituting this into the Mantel test formula yields *r*_*M*_ = 1.

Therefore, if *W* perfectly matches *D*, the empirical p-value of the Mantel test consistently returns as 1. This is equivalent to the Pearson correlation coefficient between *W* and *D*. Thus, when *W* equals *D*, the Mantel test simplifies to the Pearson correlation coefficient between the distance matrices:

$$\begin{array}{c} r_{P}=\frac{\sum_{i<j}\left(w_{ij}-\bar{W}\right)\left(d_{ij}-\bar{D}\right)}{\sqrt{\sum_{i<j}{\left(w_{ij}-\bar{W}\right)}^{2}\sum_{i<j}{\left(d_{ij}-\bar{D}\right)}^{2}}}, \end{array}$$ (4)

where *w*_*ij*_ and *d*_*ij*_ are as previously defined, and $\bar{W}$ and $\bar{D}$ denote the means of the distance matrices. The Mantel test statistic *r*_*M*_ and the Pearson correlation coefficient *r*_*P*_ are equivalent when *W* = *D*.

In this case, the null hypothesis is that there is no correlation between the two matrices, i.e., *r*_*P*_ = 0. Under the null hypothesis, the distribution of *r*_*P*_ follows a Student’s t-distribution with *n*(*n* − 1)/2 − 1 degrees of freedom, where *n* is the number of objects. In particular, the distribution is symmetric about 0, meaning that the probability of observing a value of *r*_*P*_ greater than or equal to its observed value is equal to that of observing a value of *r*_*P*_ less than or equal to its negative.

Since the empirical p-value of the Mantel test is computed as the proportion of permutations that yield a test statistic greater than or equal to the observed value plus the proportion that yields a test statistic less than or equal to the negative of the observed value, we have:

$$\begin{array}{c} p=\frac{\sum_{b=1}^{B}I\left(r_{P}^{\left(b\right)}\geq r_{P}\right)+\sum_{b=1}^{B}I\left(r_{P}^{\left(b\right)}\leq -r_{P}\right)}{2B}. \end{array}$$ (5)

Since the null distribution of *r*_*P*_ is symmetric about 0, the two sums in the numerator of the above expression are equal, and the empirical p-value is equal to:

$$\begin{array}{c} p=\frac{\sum_{b=1}^{B}I\left(r_{P}^{\left(b\right)}\geq r_{P}\right)}{B}. \end{array}$$ (6)

In other words, the empirical p-value equals the proportion of permutations that yield a Pearson correlation coefficient greater than or equal to the observed value. Since the observed value of *r*_*P*_ is a Pearson correlation coefficient between two identical distance matrices, which is always 1, the empirical p-value is 1 for any number of permutations *B*. Therefore, if *W* = *D*, the empirical p-value of the Mantel test is always 1.

#### Limitation 2

**The Mantel test p-value for permuted matrices** (*D* = *PW*) **is always 1**. Consider that *P* represents the permutation matrix that transforms matrix *W* into matrix *D*. This *n* × *n* matrix has only one 1 in each row and column, with all other entries as 0. When we multiply *W* by *P*, we get *D*.

The Mantel test statistic, denoted as *r*_*M*_, is given by:

$$\begin{array}{c} r_{M}=\frac{\sum_{i<j}w_{ij}d_{ij}}{\sqrt{\sum_{i<j}w_{ij}^{2}\sum_{i<j}d_{ij}^{2}}} \end{array}$$ (7)

Expanding the terms using the information that *D* is a result of permuting rows and columns of *W* via matrix *P*, we find that:

$$\begin{array}{c} r_{M}=\frac{\sum_{i=1}^{n}\sum_{j=1}^{n}w_{ij}\left(p_{ij}w_{ij}\right)}{\sqrt{\left(W^{T}W\right)\cdot \left(PW\right)}} \end{array}$$ (8)

Here, (*W*^*T*^*W*) represents the element-wise square of the matrix *W*, and (*PW*) stands for the multiplication of *P* by *W*. The Mantel test statistic, *r*_*M*_, can be succinctly expressed as:

$$\begin{array}{c} r_{M}=\frac{\sum_{i=1}^{n}\sum_{j=1}^{n}w_{ij}\left(p_{ij}w_{ij}\right)}{\sqrt{\left(W^{T}W\right)\cdot \left(PW\right)}} \end{array}$$ (9)

This serves as the desired expression for the Mantel test statistic, presented in terms of matrix operations. Therefore, the Mantel test statistic can be expressed as:

$$\begin{array}{c} \text{Mantel}\left(W,D\right)=\text{trace}\left(W^{T}PW\right) \end{array}$$ (10)

To prove the empirical p-value of the Mantel test is universally equal to 1, we can use the following reasoning. The Mantel test p-value is computed as:

$$\begin{array}{c} p=\frac{1}{M}\sum_{i=1}^{M}I\left(\text{Mantel}\left(W_{\pi_{i}},PW\right)\geq \text{Mantel}\left(W,PW\right)\right), \end{array}$$ (11)

where *M* is the number of permutations, $W_{\pi_{i}}$ is the matrix obtained by permuting the rows and columns of *W* according to the *i*-th permutation *π*_*i*_, and *I* is the indicator function.

Since *PW* is a permutation of *W*, the Mantel statistic will be zero, and the p-value will be 1. This is because a permutation of *W* simply rearranges the order of the pairwise distances, but does not change the actual distances between pairs of observations. As a result, the correlation between *W* and *PW* will be perfect, and the p-value will indicate that there is no evidence of a significant difference between the two matrices.

Therefore, when *W* and *D* are equal, the empirical p-value of the Mantel test is always 1. The Mantel test p-value decreases inversely with the number of permutations, *k*, as $p∼\frac{1}{k}$.

Finally, although recent publications show that the Mantel test is not affected by inflated Type I error when spatial autocorrelation affects only one variable, when investigating correlations, or when either the response or the explanatory variable is affected by spatial autocorrelation while investigating causal relationships [38], a critical limitation of the Mantel test is its reliance on distance variables, which measure the relationships between pairs of objects. The Mantel test is inherently unsuitable when applied to networks with no geographical or spatial data, such as social networks. In such cases, where the spatial arrangement or proximity of network nodes is unknown, the Mantel test becomes ineffective for assessing associations within the network. In light of the Mantel test limitations, we only focus on the Quadratic Assignment Procedure in what follows.

### Limitations of the Quadratic Assignment Procedure

**Fig 1:**
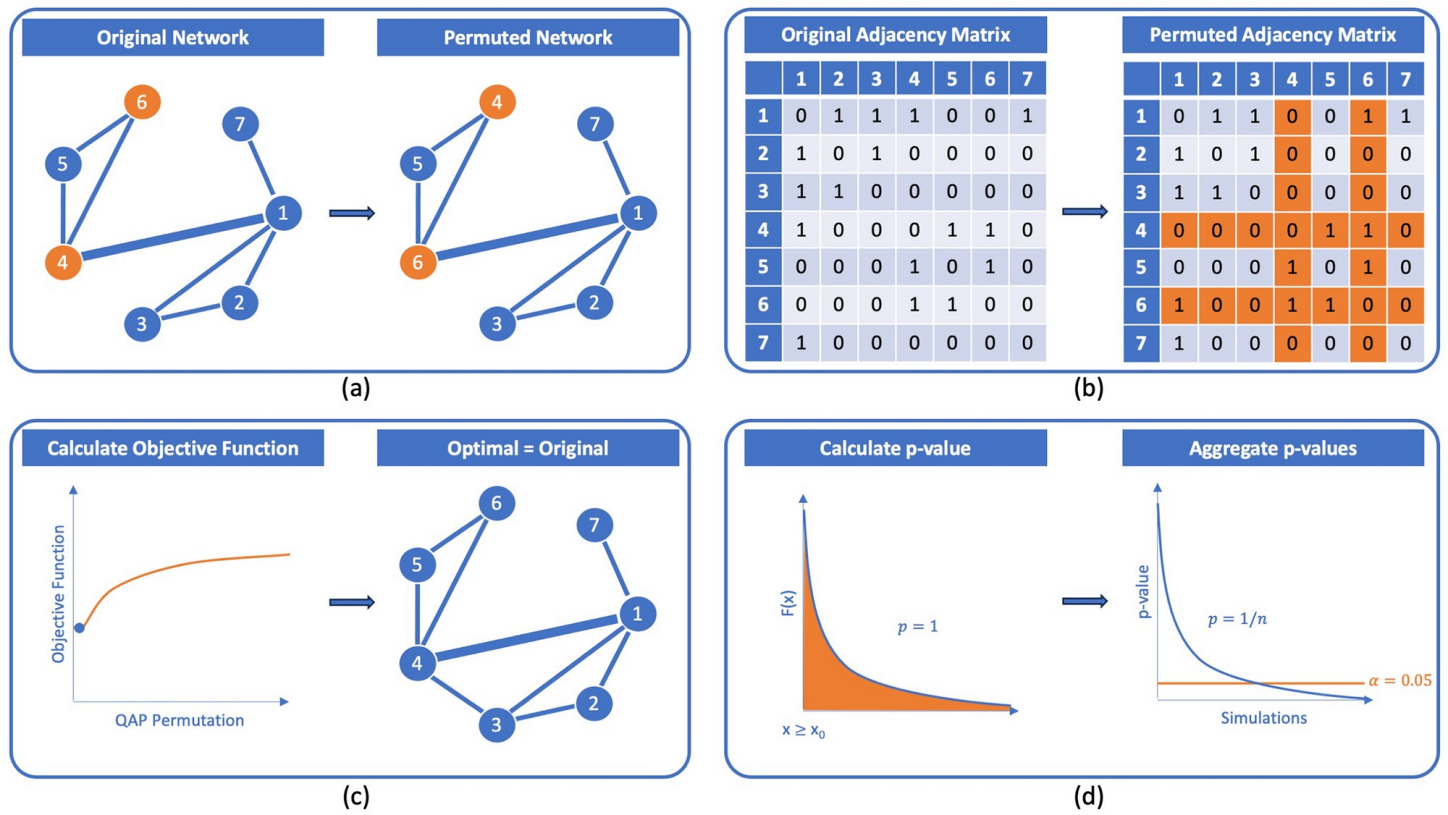
Limitations of the Quadratic Assignment Procedure scheme. (a) Two nodes of the original network are permuted, while the edges’ structure remains unchanged. (b) The adjacency matrices of the original and the modified networks are calculated. (c) The objective function is calculated using the adjacency matrices. The optimal permutation that minimizes it is the original network. (d) For each simulation, the p-value equals 1. When all p-values for the different simulations are aggregated, the p-value decays by 1/*n*.

It is worth noting that QAP also encounters limitations when comparing permutations of the same matrix [39]. The QAP objective function is invariant under row and column permutations, meaning that any permutation of the optimal assignment obtained from QAP is also optimal. Therefore, the p-value results in 1. In these cases extracting meaningful information becomes challenging [32, 34]. The implications of the following limitations are depicted in Fig 1.

#### Limitation 3

**The QAP p-value for equal matrices** (*D* = *W*) **is always 1**. If matrices *W* and *D* are equal, i.e., *D* = *W*, then the objective function of the Quadratic Assignment Procedure (QAP) simplifies to:

$$\begin{array}{c} \text{Obj}\left(W,D\right)=\sum_{i=1}^{n}\sum_{j=1}^{n}w_{ij}d_{p_{i}p_{j}}=\sum_{i=1}^{n}\sum_{j=1}^{n}w_{ij}w_{p_{i}p_{j}}=\sum_{i=1}^{n}\sum_{j=1}^{n}w_{ij}p_{ij}w_{ij}p_{ji} \end{array}$$ (12)

$$\begin{array}{c} =\sum_{i=1}^{n}\sum_{j=1}^{n}p_{ij}w_{ij}p_{ji}w_{ij}=\text{trace}\left(PWP^{T}W\right). \end{array}$$ (13)

Moreover, to minimize this objective function, *P* must be the identity permutation, as any other permutation would introduce additional terms to the trace and increase the objective. Thus, when *P* is the identity permutation, we obtain:

$$\text{Obj}(W,D)=\text{trace}\left(IWI^{T}W\right)=\text{trace}\left(W^{2}\right).$$ (14)

Since this objective is independent of the permutation matrix *P*, any permutation of the rows and columns of *W* would yield the same objective, and therefore, all permutations achieve the same objective. Consequently, any permutation is an optimal solution in this case.

Finally, when applying a permutation test to assess the statistical significance of the solution, we compare the observed objective function with the objective functions obtained under permutations. However, since all permutations achieve the same objective, the observed objective function is always as extreme or more extreme than the objective functions obtained under permutations. Therefore, the p-value obtained from the permutation test is equal to 1, indicating that the observed solution is not statistically significant.

Furthermore, if we observe the p-value *p* along the number of permutations *k*, it decreases by $p∼\frac{1}{k}$, reflecting the fact that as the number of permutations increases, the observed solution becomes increasingly likely, and its significance decreases.

#### Limitation 4

**The QAP p-value for permuted matrices** (*D* = *PW*) **is always 1**. Consider now the effect of a row permutation on the objective function:

$$\begin{array}{c} \text{Obj}\left(W^{\prime },D\right)=\sum_{i=1}^{n}\sum_{j=1}^{n}w^{\prime }ijdp_{i}p_{j}, \end{array}$$ (15)

where *W*′ is the matrix obtained by permuting the rows of *W* according to *P*. Since *P* is a permutation, the rows of *W*′ are just a reordering of the rows of *W*, so the sum in the objective function is still over the same set of elements. Therefore, the value of the objective function is the same for *W* and *W*′. Similarly, we can show that the objective function is invariant under column permutations.

When *D* is a permutation of *W*, this means a permutation matrix *P* exists, such as *D* = *PWP*^*T*^. Now, consider the objective function of the QAP with matrices *W* and *D* = *PWP*^*T*^:

$$\begin{array}{c} \text{Obj}\left(W,D\right)=\text{trace}(W^{T}PW\left(P^{T}\right)). \end{array}$$ (16)

Since *P* is a permutation matrix, *P*^*T*^ is also a permutation matrix. Therefore, this objective function is equivalent to the original objective function for matrices *W* and *D*.

In addition, if *D* is a permutation of *W*, and *P* is the permutation matrix such that *D* = *PWP*^*T*^, any optimal solution to the QAP with matrices *W* and *D* = *PWP*^*T*^ corresponds to an optimal permutation matrix *P*′. Since *P*′ represents the optimal assignment for matrices *W* and *D* = *PWP*^*T*^, applying the inverse permutation *P*^−1^ (which is also a permutation matrix) to *P*′ will yield an optimal assignment for matrices *W* and *D*. Therefore, the optimal solution for matrices *W* and *D* = *PWP*^*T*^ can be obtained by applying the corresponding permutation *P* to the rows and columns of *W*.

As established above, the objective function Obj(*W*, *D*) is invariant under row and column permutations of *W* and *D*. Therefore, any permutation of the optimal assignment obtained from the QAP yields the same objective. Consequently, all permutations achieve the same objective, and the p-value obtained from a permutation test is always 1 when *D* is a permutation of *W*.

## Network randomization methods

While permutation tests like the Mantel test and QAP are valuable tools in network analysis, they may not be optimal for detecting minor differences in network topologies. Alternative network randomization methods, such as degree-constrained link shuffling or connected degree-constrained link shuffling, offer more robust null models that can better capture subtle variations in network structures, enhancing the accuracy and reliability of statistical inferences in network analysis [40].

In the statistical inference realm, the term *null model* has conventionally been used to describe the likelihood of an observation occurring by chance. However, this term might inaccurately suggest the absence of relevant patterns in the system under study. Gauvin et al. [40] propose the term *reference models* as a more appropriate alternative to null models. The term reference underscores the idea that observations are not being compared to a completely random scenario devoid of predictable patterns, but rather to a system where certain features of interest are retained while others are randomized. In what follows, we will refer to these models as reference models.

In network analysis, the search for robust statistical inferences often depends on the efficacy of reference models in capturing the peculiarities of network structures. The *degree-constrained link shuffling* is a widely-used technique for randomizing networks while preserving their degree distribution, also known as Maslov-Sneppen method [41]. Its fundamental idea is to keep node degrees while randomly reshuffling their links [42]. However, it may generate disconnected networks, particularly in sparse networks. To overcome this limitation, [40] presented *connected-degree constrained link shuffling* to ensure the connectedness of the resulting networks. These techniques are strictly equivalent for a large number of swaps. Both methods can be considered as specific versions of the *configuration model* [43], which generates random graphs based on a given degree sequence. However, these techniques are not grounded on *Markov Chain Monte Carlo* (MCMC) methods, then we cannot generate meaningful *p*-values to check whether there are subtle differences between reference models and the original networks that occur by random chance or there are significant differences between them.

The use of MCMC algorithms with edge swap or rewiring techniques has been a prevalent method for generating randomized networks [44]. A MCMC approach involves iteratively swapping pairs of connections within a network until a well-randomized structure is achieved. The process of edge swapping within MCMC schemes has been shown to produce new, quasi-independent network samples. Specifically, double edge-swap MCMC methods have been highlighted for their ability to uniformly sample from various graph spaces given sufficient time [45]. When analyzing p-values, we must create multiple non-significant randomized versions of the original network, enabling subsequent comparisons. In this context, the MCMC approach offers the advantage of producing several randomized versions, each with distinct topological features but statistically comparable to the original network, allowing for comprehensive comparative analysis. While MCMC algorithms with edge rewiring techniques offer valuable advantages for generating randomized networks, they also come with limitations, including computational complexity, potential biases, and limited flexibility [46].

Alternative network randomization approaches, such as Exponential random graph models (ERGMs) [47, 48], provide insights into the entire network structure significance by permuting both nodes and edges, capturing node-level characteristics and the network’s underlying structure. However, ERGMs have limitations: they are computationally intensive, require careful model specification to avoid bias, are prone to overfitting due to their inherent parameters complexity, can be complex to interpret, need adequate data for reliable estimates, and, in general, pose challenges in model selection [49, 50].

## Proposed alternatives for network randomization

Incorporating edges’ rewiring techniques in permutation tests allows for examining the significance of network structure by altering edges while keeping nodes fixed. This expands the scope of permutation tests and enables the assessment of the impact of edge rearrangement on network properties. Rewiring can be considered an example of local permutation in network analysis because it involves the local rearrangement of edges while keeping the overall node configuration fixed. By doing so, researchers can explore alternative network configurations [12].

Permutation tests modify the network by changing the node attributes but keeping the edges’ properties unchanged. Therefore, nodes change their roles, however, the overall network structure remains the same. As a result, node permutation does not identify similarities in topology but explores the relation between node attributes and their network location. Conversely, the shuffle procedure randomly reorders the edges, leading to a completely reorganized connection distribution while maintaining the node attributes. Shuffling results in nodes keeping their initial attributes but changing their relationships, causing the dismantling of the original core nodes. This random reorganization leads to significant information loss and directly affects the edges’ distribution, which impacts the statistical significance tests. Therefore, we exclude permutation and shuffle procedures as viable randomization alternatives.

To assess the statistical significance of network topologies, we must compare it to a modified version of the original structure (our reference model). However, there are different ways to modify a network topology. Here, we compare the two proposed MCMC alternatives: *random rewiring* and *controlled rewiring*. A comprehensive comparison of the alternatives is presented in Table 1.

**Table 1:** Comparison between alternatives when changing the network topology.

Alternative	Nodes	Edges	Structure
Random Rewiring	Remain the same	Randomly swapped by pairs	Original core nodes are kept but reconnected to other nodes. The original structure is strongly modified.
Controlled Rewiring	Remain the same	Swapped by pairs according to a metric	Original core nodes are kept but reconnected to other nodes. The original structure is subtly modified.

### Random rewiring

The *random rewiring* alternative (Algorithm 1) described in [51] uses *edge-swapping Markov Chain Monte Carlo* methods to modify connections by keeping the degree distribution and loopless nodes as constraints. Random rewiring changes the edges, randomly selecting two distinct edges (*v*_*i*_, *v*_*j*_) and (*v*_*k*_, *v*_*l*_) from the network. These chosen edges are then swapped to form either (*v*_*i*_, *v*_*k*_), (*v*_*j*_, *v*_*l*_), or (*v*_*i*_, *v*_*l*_), (*v*_*j*_, *v*_*k*_) at random. This process may introduce self-loops or multiple edges. However, in this research, we only consider simple graphs, i.e., there are no self-loops.

**Fig 2:**
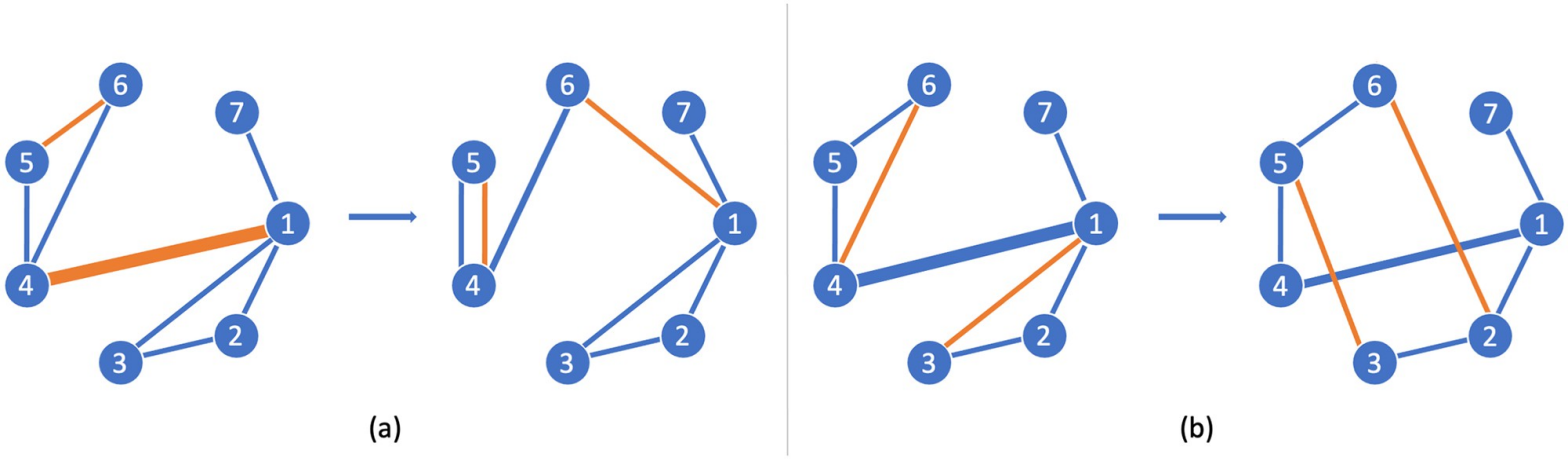
Randomization alternatives scheme. In all examples, the thickness of an edge represents its betweenness centrality. (a) The random rewiring alternative swaps the edges by randomly selected pairs while nodes remain the same. The edges betweenness centrality is still altered. (b) Finally, in the controlled rewiring alternative, the edges to be rewired are selected according to a metric and the edge betweenness centrality is less altered.

For instance, in Fig 2(a), the rewiring algorithm randomly selects edges (*v*_5_, *v*_6_) and (*v*_1_, *v*_4_), then it swaps the edge-pair into (*v*_1_, *v*_6_) and (*v*_4_, *v*_4_), as displayed. Consequently, the network’s degree distribution remains constant, meaning every single node keeps its number of connections.

Rewiring modifies the network structure while keeping some topological constraints. The rewired network preserves some statistics, such as degree distribution. Therefore, rewiring keeps some properties of the original network, maintaining those highly connected nodes with the same number of outgoing connections but changing their destinations. To this extent, random rewiring allows us to analyze the change in topology without entirely breaking the existing structures, only changing link destinations.

**Algorithm 1** Random rewiring

**Require**: Graph *G* = (*V*, *E*), number of rewirings *n*

**Ensure**: Rewired graph *G*′ = (*V*, *E*′)

 **for** *k* ← 1 to *n* **do**    ▹ *n* ≤ |*G*.*E*| where |*G*.*E*| stands for the number of edges

$\left(v_{a},v_{b})\leftarrow U(0,|G.E|\right)$ ▹ Randomly choose the first edge to rewire

$\left(v_{c},v_{d})\leftarrow U(0,|G.E|\right)$ ▹ Randomly choose the second edge to rewire

  **if** a≥U(0,1) **then**

   *G*.*E*(*v*_*a*_, *v*_*b*_), *G*.*E*(*v*_*c*_, *v*_*d*_) ← (*v*_*a*_, *v*_*c*_), (*v*_*b*_, *v*_*d*_)      ▹ Rewire operation

  **else**

   *G*.*E*(*v*_*a*_, *v*_*b*_), *G*.*E*(*v*_*c*_, *v*_*d*_) ← (*v*_*a*_, *v*_*d*_), (*v*_*b*_, *v*_*c*_)      ▹ Rewire operation

  **end if**

 **end for**

 **return** *G*

**Example**. *Following the example in* Fig 2(a), *after random rewiring, a manager will still be a manager but connected to different employees. Although the number of connections of each employee remains equal, their centrality within the organization considerably changes*.

### Controlled rewiring

We introduce a new approach called *controlled rewiring* (Algorithm 2) alongside the random rewiring technique to enhance the alternatives’ comprehensiveness. In the controlled rewiring alternative, the connections between edges are modified similarly to random rewiring, but, instead of selecting edges randomly, we base the selection on their betweenness centrality. We opt for edge betweenness centrality because allows us to sort edges by considering their centrality, and it is not inherently limited to symmetrical networks.

**Algorithm 2** Controlled rewiring

**Require** Graph *G* = (*V*, *E*), number of rewirings *n*, number of bins *b*

**Ensure**: Rewired graph *G*′ = (*V*, *E*)

 *E*′ ← sort(*G*.*E*)         ▹ Sort edges by their betweenness value

 *B* ← binarize(*G*.*E*,*b*)     ▹ Group edges in smaller groups by their betweeness

 **for** *b*_*i*_ ∈ *B* **do**          ▹ Traverse resulting sorted bins

  **for** *k* ← 1 to |*b*_*i*_|/2 **do**    ▹ |*b*_*i*_| stands for the number of edges in the bin *b*_*i*_

$\left(v_{a},v_{b})\leftarrow U(0,|b_{i}|\right)$ ▹ Randomly choose the first edge to rewire

$\left(v_{c},v_{d})\leftarrow U(0,|b_{i}|\right)$ ▹ Randomly choose the second edge to rewire

   **if** a≥U(0,1) **then**

    *G*.*E*(*v*_*a*_, *v*_*b*_), *G*.*E*(*v*_*c*_, *v*_*d*_) ← (*v*_*a*_, *v*_*c*_), (*v*_*b*_, *v*_*d*_)      ▹ Rewire operation

   **else**

    *G*.*E*(*v*_*a*_, *v*_*b*_), *G*.*E*(*v*_*c*_, *v*_*d*_) ← (*v*_*a*_, *v*_*d*_), (*v*_*b*_, *v*_*c*_)      ▹ Rewire operation

   **end if**

  **end for**

 **end for**

 **return** *G*

Traditionally, network literature has assumed full symmetric networks when using betweenness centrality metrics. However, some recent studies use edge betweenness centralities to identify critical edges [52], that serve as bridges or bottlenecks in the network, or to identify community structures in social and biological networks [53], or to test the efficiency of new topological metrics [54], regardless of whether the relationships in the network are bidirectional or asymmetrical. These studies highlight that edge betweenness centrality can be utilized in directed networks, considering the directionality of edges. This adaptation allows for the analysis of the importance of edges in facilitating communication or flow within directed networks, providing valuable insights into the structural significance of edges in asymmetrical network models. Therefore, in scenarios where the directional relationships between nodes are crucial, edge betweenness centrality can still offer meaningful centrality assessments in asymmetrical networks by considering the specific characteristics of directed edges.

Initially, we divide edges into smaller groups, and the rewiring process begins with the most outlying group, gradually progressing toward the most central ones. Within each group, random rewiring is applied exclusively to the edges. This approach enables precise control over changes in the network structure, ensuring a consistent degree of distribution and a more stable centrality of the edges.

For example, in Fig 2(b), the controlled rewiring algorithm selects edges (*v*_4_, *v*_6_) and (*v*_2_, *v*_3_) and swaps them to form (*v*_3_, *v*_5_) and (*v*_2_, *v*_6_), respectively, as depicted. Despite the slight modification in the network’s topology, the degree distribution remains unchanged, meaning that each node maintains its number of connections. This controlled rewiring technique also enables us to retain central edges within the network, albeit in a more gradual manner where they are exchanged or replaced as shown below.

**Example**. *Following the example in* Fig 2(b), *after controlled rewiring, connections are swapped only between nodes of similar centrality. Therefore, a manager will still be a manager and the company’s hierarchy is only subtly altered*.

### More gradual changes in average edge betweenness centrality

Here, we aim to analyze how the total absolute change in average edge betweenness centrality is minimized in controlled rewiring compared to random rewiring. The controlled rewiring algorithm takes a graph *G* = (*V*, *E*), several rewirings *n*, and bins *b* and rewires the graph, ensuring that specific properties, such as edge betweenness centrality, are preserved as much as possible. Since many topological metrics values are node or edge-wise, to evaluate rewiring impact, we should analyze their corresponding distribution. Specifically, we study the changes in the average and standard deviation of edge betweenness centrality. The average edge betweenness is given by $\bar{B}\left(E\right)=\frac{1}{\left|E\right|}\sum_{e\in E}\;B\left(e\right)$. Let *E* be sorted such that $B\left(e_{1}\right)\leq B\left(e_{2}\right)\leq \ldots \leq B\left(e_{\left|E\right|}\right)$. Then, following the controlled rewiring algorithm, we divide *E* into *n* bins: *b*_1_, *b*_2_, …, *b*_*n*_. Afterward, for each bin *b*_*i*_, edges within each bin are rewired.

Although the original edges in *b*_*i*_ have similar betweenness centrality values ($B\left(v_{a},v_{b}\right)\approx B\left(v_{c},v_{d}\right)$), new resulting edges (*v*_*a*_, *v*_*c*_) and (*v*_*b*_, *v*_*d*_) (or (*v*_*a*_, *v*_*d*_) and (*v*_*b*_, *v*_*c*_)) may have distinct betweenness centrality values. That is because the rewiring can create or eliminate some shortest paths, thus affecting rewired edges betweenness centrality. However, we have empirically observed that average edge betweenness centrality changes are lower after controlled rewiring than after random rewiring. Therefore, $|\Delta {\bar{B}}_{\text{CR}}\left(E\right)|\leq |\Delta {\bar{B}}_{\text{RR}}\left(E\right)|$, being $\Delta {\bar{B}}_{\text{CR}}\left(E\right)$ the average betweenness variation for the controlled rewiring, and $\Delta {\bar{B}}_{\text{RR}}\left(E\right)$ for the random rewiring. Nevertheless, a single application of network rewiring does not fully explore the solution space. In contrast, Markov Chain Monte Carlo (MCMC) methods offer a probabilistic framework that can significantly enhance the effectiveness of rewiring algorithms by providing robust mechanisms for sampling and exploring several solution spaces. By integrating MCMC into the provided rewiring algorithms, researchers can explore diverse configurations of edge rewiring while targeting specific network metrics, such as rewiring edge betweenness centrality. Additionally, applying MCMC provides insights into the robustness and reliability of network rewiring algorithms by observing metrics mean and standard deviation distributions over the simulations to analyze the range of possible network evolutions after controlled or random rewiring. To this end, we design an MCMC experiment that repeats the same experiment for 100 simulations, each making enough iterations to swap all the edges in the network only once.

**Example**. *In* Fig 2(b), *we apply controlled rewiring by swapping the most peripherical edges*, (*v*_4_, *v*_6_) *and* (*v*_2_, *v*_3_), *displayed in orange. In this example, edge betweenness values are relatively high for certain edges, particularly* (*v*_1_, *v*_4_) *and* (*v*_1_, *v*_7_). *These high values indicate that these edges are critical for maintaining the shortest paths within the network. Comparing the original and rewired edge betweenness centrality values, we can draw the following conclusions: the original graph has an average edge betweenness of* 5.00. *Note that the igraph edge betweenness function does not normalize the values of edge betweenness as other packages do. After rewiring edges, the resulting graph exhibits an average mean edge betweenness of* 4.75. *Contrarily, the rewired graph generated through random rewiring* (Fig 2(a)) *has a higher average edge betweenness* (5.63), *indicating that the rewiring has introduced new critical edges or increased the importance of existing ones, particularly edges like* (*v*_1_, *v*_6_) *and* (*v*_4_, *v*_6_).

**Fig 3:**
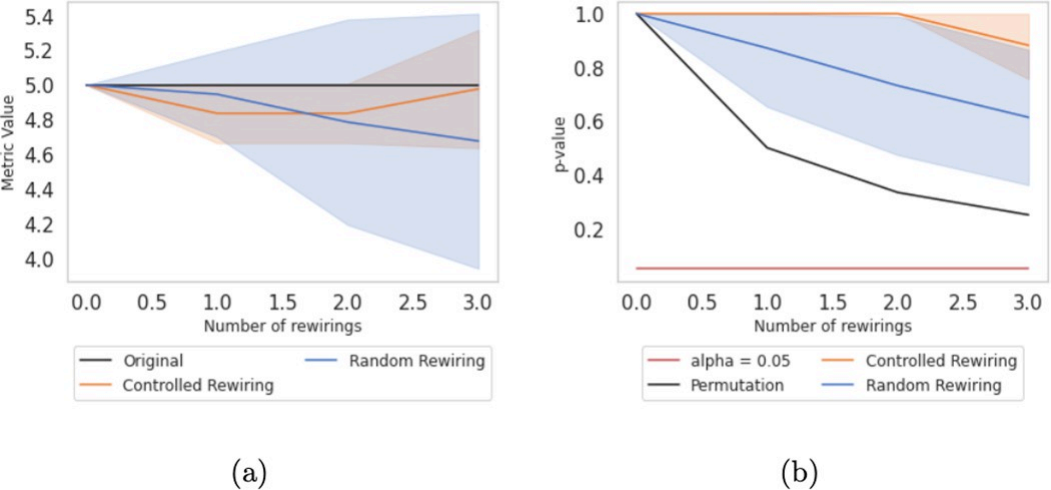
(a) Average edge betweenness centrality of the Fig 2 network example for 100 simulations along three controlled rewirings (orange) and three random rewirings (dark blue). Means are plotted in lines, and the standard deviation in shadowed areas (b) p-value by performing the Expanded Quadratic Assignment Procedure of the Fig 2 network example for 100 simulations along different numbers of rewirings. Means are plotted in lines, and the standard deviation is in shadowed areas.

*To check if these differences are significant, we have executed 100 simulations using the controlled and random rewiring methods and computed their corresponding p-value using the Expanded Quadratic Assignment, explained in the next section. We depict the metric differences in* Fig 3(a), *where we observe that controlled rewiring appears to make more minor changes to the average edge betweenness. When analyzing the p-values in* Fig 3(b), *we observe that controlled rewiring generates models that do not present significant differences compared to the original network. Although the models generated using random rewiring are also not significant, we detect that the p-value decreases faster when using this alternative. However, we want to stress that in this small example, the significance of the difference is influenced by the network size*.

**Fig 4:**
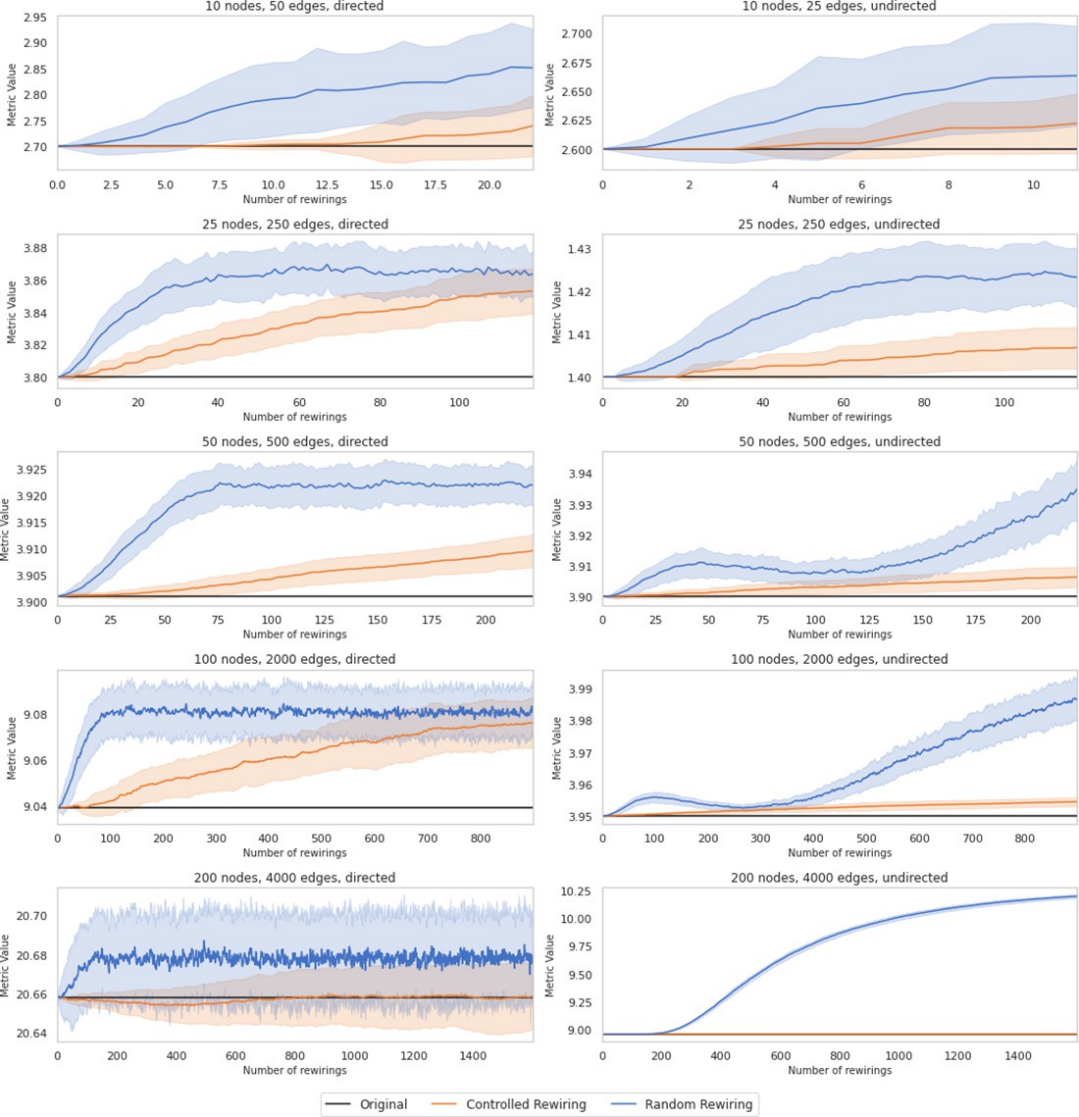
Average edge betweenness centrality of different random Power Law network for 100 simulations along different numbers of rewirings. Means are plotted in lines, and the standard deviation is in shadowed areas.

Before testing the described alternatives in real data sets, we benchmark them against several Power Law networks of different sizes generated at random. The primary rationale is that, after performing controlled rewiring, the average edge betweenness centrality changes should be more gradual than after random rewiring. To statistically validate this idea, we generate the random networks according to the parameters of the well-known Enron Email Data set [55]. By using the function *Fit* from the Python *powerlaw* package [56, 57], we retrieve the exponential parameters of that network for in- and out-degree distribution [58, 59]. Later, with the function *Static_Power_Law* of *igraph*, we have generated a series of random Power Law networks. We repeated the same process for various network sizes to check possible divergent results caused by network size. However, all trials presented similar behaviors even though the results may vary depending on the network density and whether it is directed or undirected. Therefore, for brevity, we only include a few of them in Fig 4. In black, we depict the original edge betweenness centrality, the blue line corresponds to the average edge betweenness centrality for 100 simulations along different numbers of random rewirings, and finally, the orange line pots the average edge betweenness centrality for controlled rewirings. The shadowed areas represent the standard deviation after 100 simulations.

The evolution of the average edge betweenness centrality versus the number of rewirings illustrates two distinct behaviors: controlled rewiring induces moderate variations in average edge betweenness centrality. In contrast, random rewiring leads to more sparse values. By comparing both lines, it is possible to identify that the induced changes in controlled rewiring imply a more gradual variation across the different graph configurations when we increase the number of rewirings.

Besides, we want to highlight that the impact of graph configuration on rewiring strategies is notable. For example, when comparing the observed metric changes in left (directed) and right (undirected) columns in Fig 4, random rewiring, for example, often produces early sharper increases in edges betweenness centrality for directed graphs even with few rewired edges.

## Expanding the statistical tests

The main contribution of this research is the combination of rewiring methods with the expansion of the Quadratic Assignment Procedure (QAP) to assess the statistical significance of network topologies. The expanded version of this statistical test integrates a new approach, incorporating modifications to the adjacency matrix based on the previously explained methods. This expanded algorithm aims to calculate the resulting topological metrics, the optimal QAP assignment matrix, and its respective p-values while considering the changes made to the network structure.

Before expanding the QAP, we also expanded the Mantel test, as explained in the Appendix, by randomly modifying the values in the adjacency matrix based on the chosen alteration method (random or controlled rewiring). The algorithm returns the correlation coefficients, the p-value, and the recalculated metric measures. However, when comparing a modified network to the original one, the initial correlation coefficient will always be 1, and the changed coefficient will always be 0, as discussed in the limitations section. Therefore, the Expanded Mantel test does not provide more exhaustive results, but further improvements can be achieved by expanding the Quadratic Assignment Procedure (QAP).

The **Expanded Quadratic Assignment Procedure** (EQAP) is an algorithm that iterates *s* times to find the optimal assignment matrices *O*, compute their corresponding p-value *p*, and determine the metric measures M based on the adjacency matrix *A* of *G*. The EQAP (Algorithm 3) starts by calculating the optimal assignment matrix applied to the matrix *A* and itself. Then, the adjacency matrix *A* undergoes random changes within each iteration according to the specified Δ method. Then, the selected M metric is computed and stored. After that, the optimal assignment cost function is applied to the modified matrix *A*′ and *A*, determining the optimal assignment value *o*_*k*_. If *o*_*k*_ is greater than or equal to the initial objective function value *o*, the *counter* is incremented by 1. This enables the comparison of objective function values between the original and modified matrices. Upon completion of all iterations, the p-value is obtained by dividing *counter* by *n*. Finally, the algorithm returns the optimal assignment matrices *O*, their p-value *p*, and the metrics *M*.

**Algorithm 3** Expanded Quadratic Assignment Procedure (EQAP)

**Require**: Adjacency matrix *A*, modification algorithm Δ, number of changes *n*, topological metric M, number of simulations *s*

**Ensure**: Recalculated metrics *M*, Optimal cost objective functions *O*, *p*-value *p*

 o ← Cost(*A*,*A*)    ▹ Compute the initial value of the cost objective function

 counter ← 0      ▹ Initialize counter

 *M*, *O* ← [], []     ▹ Create empty lists

 **for** *k* ← 1 to *s* **do**    ▹ Number of simulations

  *A*′ ← Δ(*A*, *n*)   ▹ Apply the number of changes

  *M*.add(M(*A*′))  ▹ Add the recalculated topological metric

  o_k_ ← Cost(*A*,*A*′)  ▹ Compute the resulting cost objective function with the modified matrix

  *O*.add(o_k_)    ▹ Add the recalculated objective function

  **if** o_k_ ≥ o **then**   ▹ Consider only simulations with a resulting cost function greater or equal than o

   counter ← counter + 1

  **end if**

 **end for**

$p\leftarrow \frac{\text{counter}}{s}$ ▹ Calculate the *p*-value

 **return** *O*, *p*, *M*

**Fig 5:**
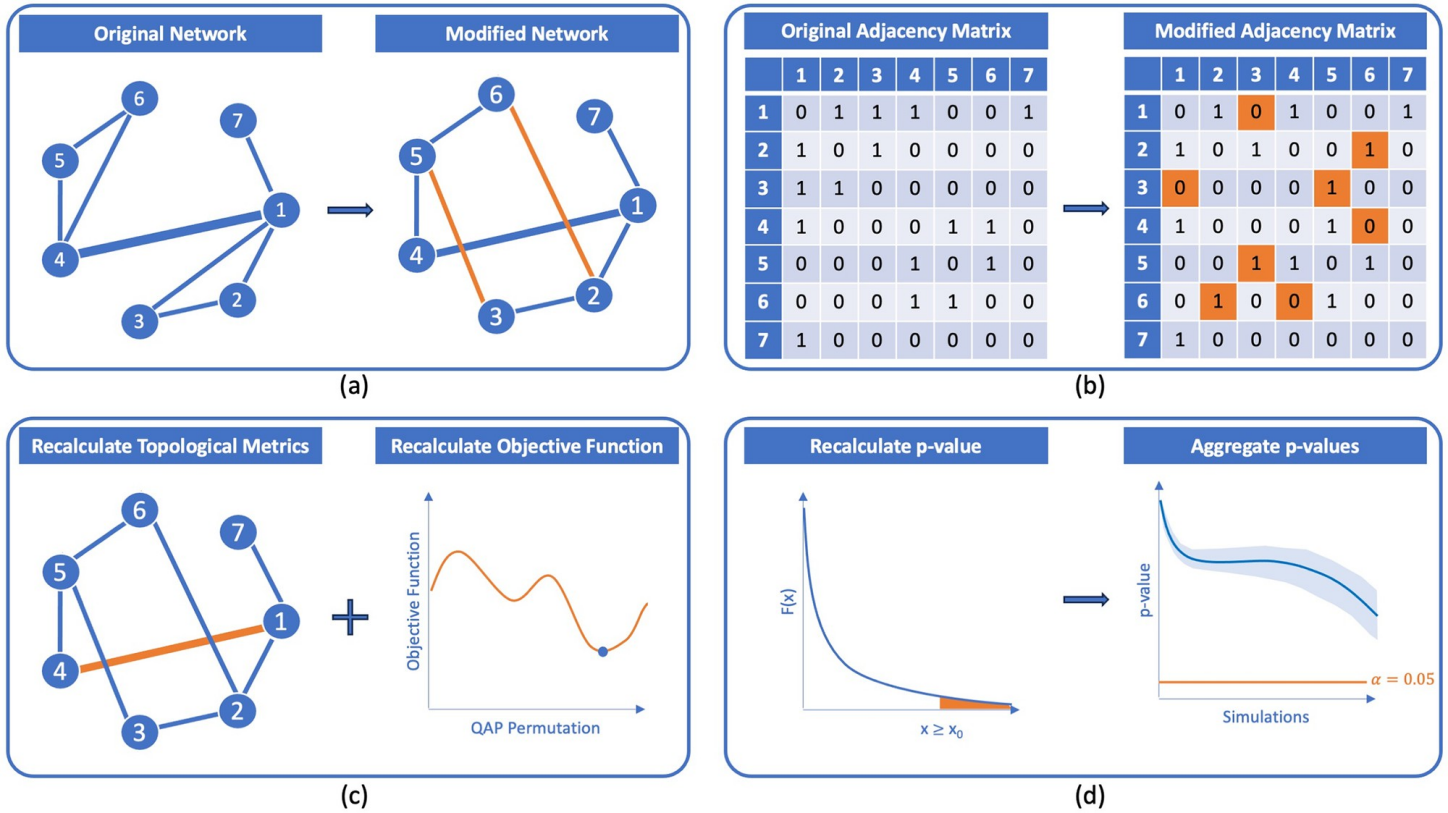
Expanded Quadratic Assignment Procedure scheme. The following pipeline is repeated for several simulations and modifications: (a) The first step is to modify the original network with the explained methods in Fig 2, i.e., random rewiring or controlled rewiring. In this scheme, we show the controlled rewiring example. (b) The second step is to build the adjacency matrices of the original and the modified networks. We will use them to calculate the objective function. (c) The third step is to measure the described topological metrics to assess the impact of the modifications and the objective function for both networks. (d) Finally, we can calculate the p-value by comparing the minimum of the objective function of the modified network to the value for the original one. All p-values for the different simulations are aggregated at last to build the results chart.

An illustrative scheme about the functioning of EQAP is depicted in Fig 5. Researchers can explore the impact of different rewiring mechanisms on network properties, enhancing their understanding of the underlying network structure and its significance [13], as summarized in Table 2.

**Table 2:** Comparison of the capabilities between the existing statistical test and the expanded methods.

	Mantel Test	QAP	EQAP
Assesses similarity or dissimilarity between networks	✓	✓	✓
Compares networks based on structure	✓	✓	✓
Evaluates consistency of network metrics	✓	✓	✓
Examines relationships with external variables	✓	✓	✓
Provides robust method for testing association	X	✓	✓
Allows altering edges while keeping nodes fixed	X	X	✓
Generates reference models for significance assessment	X	X	✓
Explores impact of different rewiring mechanisms	X	X	✓

### More gradual changes in statistical significance

Here, we benchmark the EQAP against some random networks before testing it with real data sets. The main rationale is that the new statistical test should not detect any statistical significance in network topologies after controlled rewiring. In contrast, it might be possible to detect it after random rewiring.

**Fig 6:**
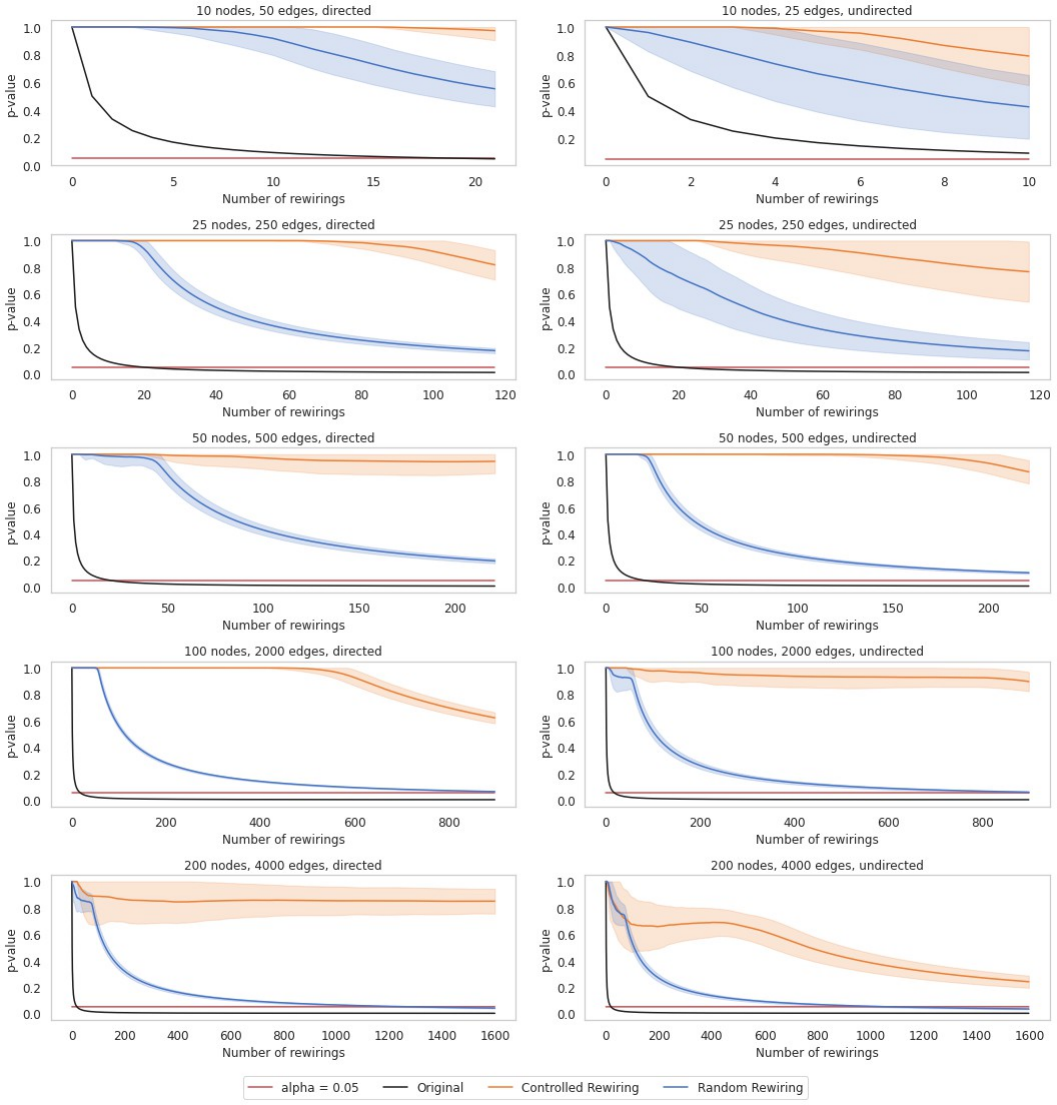
p-value by performing the Expanded Quadratic Assignment Procedure of different random Power Law networks for 100 simulations along different numbers of rewirings. Means are plotted in lines, and the standard deviation is in shadowed areas.

Using the same synthetic networks as in the previous section, Fig 6 displays a detailed comparison of rewiring strategies focused on the stability of the p-value, which represents a probability of obtaining random results at least as extreme as the observed results, under the assumption that the null hypothesis is true. We observe that controlled rewiring helps to maintain certain graph properties, leading to a more stable p-value. In contrast, random rewiring causes significant fluctuations in graph properties, resulting in a more rapid decay of the p-value. These decreases reflect the unpredictable nature of random changes, which correspond to significant network property alterations.

Due to its constricted changes, controlled rewiring offers greater predictability and reliability, making it easier to anticipate the effects of rewiring. This strategy is ideal for scenarios where the integrity of the network’s structure needs to be preserved, such as in reference model analysis. Conversely, random rewiring provides less predictability and reliability, making it suitable for exploratory contexts where the goal is to understand the effects of random changes or generate diverse graph configurations, such as in certain simulations.

One may consider that the p-value could reach a significance level below 0.05 by adding more rewirings. However, our analysis intends to demonstrate the behavior of the p-value with a controlled amount of rewiring, ensuring that each edge is altered at most once to maintain the integrity of the network’s structure. Excessive rewiring, which would significantly change the network, is beyond the scope of our current analysis.

## Numerical experiments

In what follows, we bridge the gap between theory and practice by using the Enron-Email and the UK Faculty datasets and computing the statistical significance of the modified versions and some selected topological metrics. Previously, we tested the expanded statistical tests with a synthetic Reference Model to empirically validate the utility of the proposed methodology.

Our implementation is coded in Python 3.10. For reproducibility, we have created an open-source library on GitHub [60]. This library combines different functions of the *Python igraph* package to assess the statistical significance of the topological descriptors introduced before. Moreover, we added a calculation of the *p*-values to compare the obtained metrics to the initial ones to complete the analysis. Finally, the library also includes functions to recreate the figures of this article.

### Datasets

The **Enron-Email** dataset is a collection of 520,900 emails between 184 users published by the US Department of Justice [61]. It is a temporal record of internal communication within an organization dealing with a dire crisis that threatens its existence. Interpersonal contact increased and spread throughout the network during the crisis because previously isolated personnel started talking to each other, avoiding formal communication channels. Since the dataset collected single emails, two nodes may have multiple-edge connections. For this reason, we used function *simplify* from *igraph* to remove self-loops and multiple edges. This network consists of 184 nodes and 3,010 edges.

The **UK Faculty friendship** network consists of 81 nodes and 817 edges, representing the personal friendship among the faculty members of a UK university. This social network represents tie strength between individuals with directed and weighted connections. Relationships were measured with a questionnaire, where the items formed a reliable scale [62].

### Statistical significance

**Fig 7:**
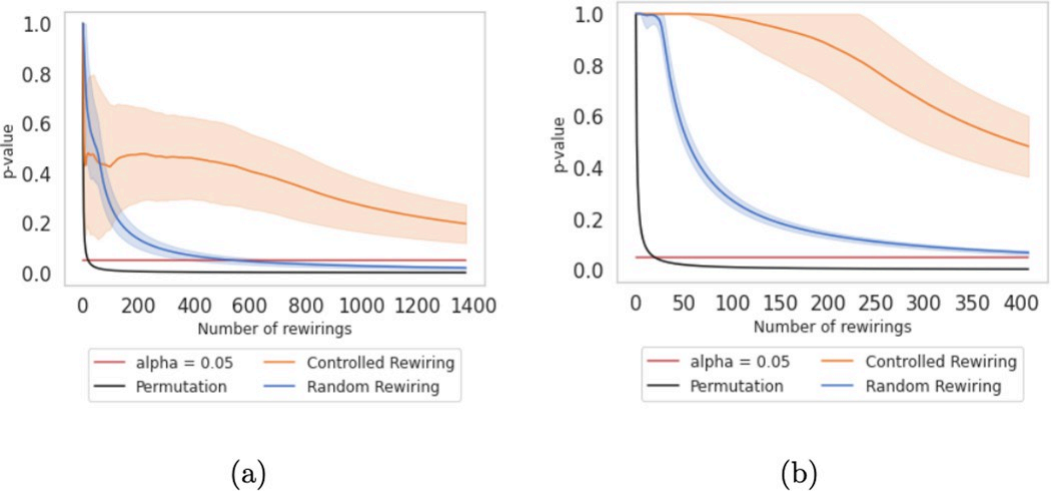
p-value by performing the Expanded Quadratic Assignment Procedure for the Enron-Email network (a), the UK Faculty network (b) networks. For both graphs, the solid line represents the mean after 100 simulations, and the shadowed band shows the standard deviation.

In Fig 7, we observe the p-values obtained by performing the Expanded Quadratic Assignment Procedure with the different rewiring alternatives. We display the evolution of p-values for 100 simulations, along with 1,500 rewirings for each. In black, we display the 1/*n* curve. Despite the permutation curve being useless to protect researchers against Type I errors, we depict it in the graphs for ease of results’ interpretation. In blue, we show the p-values after random rewiring. In orange, after controlled rewiring.

The p-values for the EQAP after 1,500 rewirings on the Enron-Email and 400 on the UK Faculty networks are displayed in Fig 7(a) and 7(b), respectively. It is worth noting that the number of rewirings is contingent on the number of edges within the chosen network since it entails modifications within a specific set of edges *E*. Since both networks contain different amounts of edges, we have set the corresponding number of alterations proportional to the number of edges.

The p-value obtained from the EQAP quantifies the evidence against the null hypothesis, which states no relationship or similarity between the matrices. The implications of rewiring, whether random or controlled, on the EQAP p-value depend on how the rewiring procedure affects the similarity between the matrices being compared. After random rewiring, the p-value obtained from the EQAP quickly decreases to a significance level below 0.05. This rapid decrease in p-value makes the random rewiring alternative sensitive when randomly switching central edges. Again, the generated reference models do not prevent researchers from rejecting a true null hypothesis.

On the contrary, controlled rewiring modifies the network based on edge betweenness centrality. In this scenario, the p-value never reaches a significance level below 0.05. The p-value obtained from the EQAP after applying the controlled rewiring increases or decreases depending on how the rewiring impacts the similarity between the original network and the reference models. For the Enron-Email network, we observe a significant decrease in the first changes, followed by a smooth increase and, later, another decrease when changes affect the most central nodes, never reaching significant differences. For the UK Faculty network, the p-value is very close to 1 when rewiring the more peripherical edges, whereas it halves its value when reaching the most central ones.

**Fig 8:**
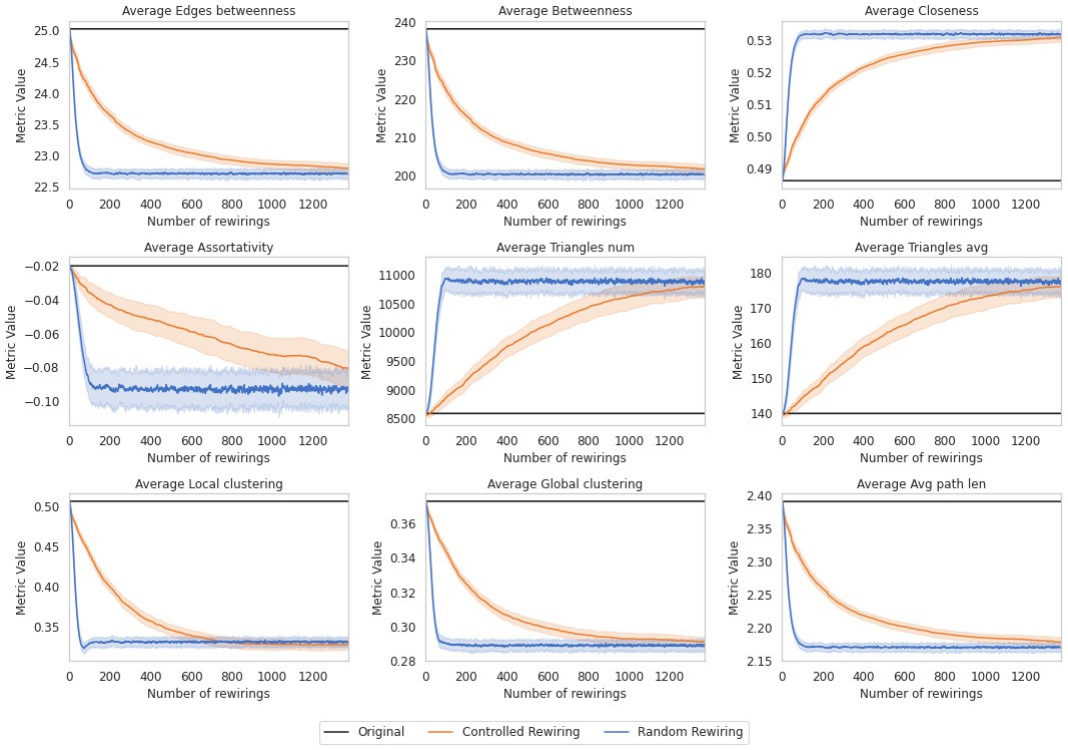
Topological metrics of the Enron-Email network for 100 simulations along 1,500 controlled rewirings (orange), 1,500 random rewirings (dark blue). Averages are plotted in lines and the standard deviation in shadowed areas.

**Fig 9:**
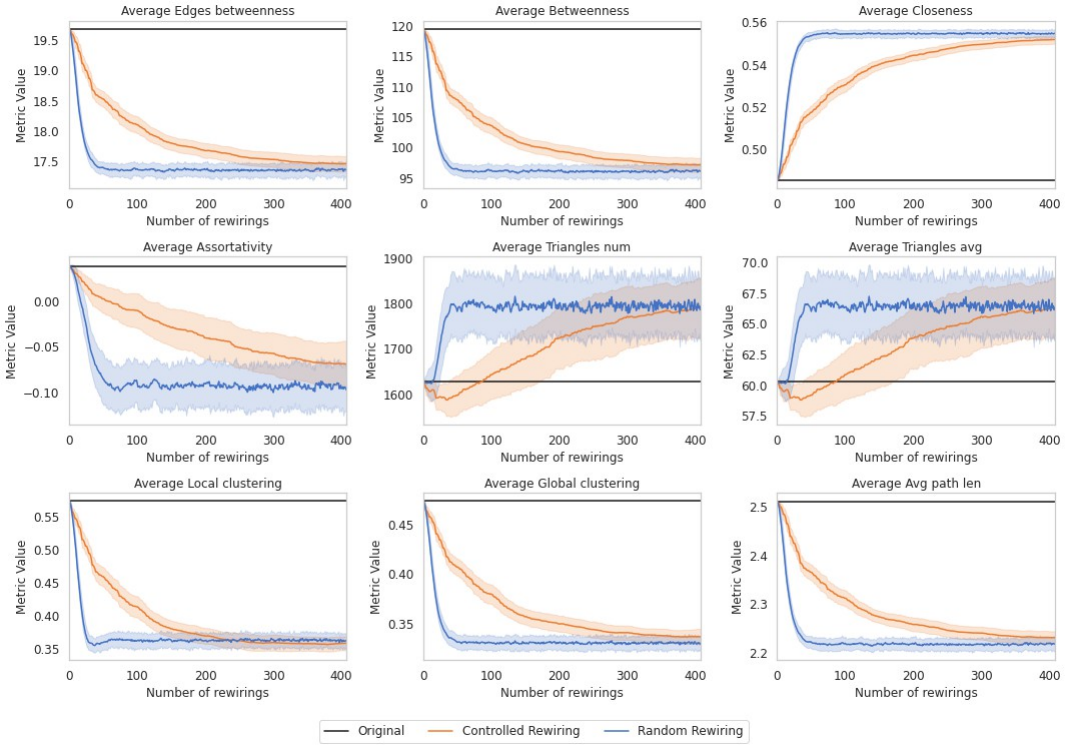
Topological metrics of the UK Faculty network for 100 simulations along 400 controlled rewirings (orange), 400 random rewirings (dark blue). Averages are plotted in lines and the standard deviation in shadowed areas.

These results are also consistent with the behavior observed in the topological metrics, as we will show in Figs 8 and 9.

### Topological metrics of real data

After testing our method on random synthetic networks, we repeated the analysis with the Enron-Email and the UK Faculty networks. In Fig 8, we display the metrics of the Enron-Email network for 100 simulations, along with 1,500 rewirings for each. We create a chart for each metric using the two randomization alternatives. In black, we display the metric results for the original network, corresponding to the metric for the network after permutations. In dark blue, we show the results of the metrics after random rewiring. In orange, after controlled rewiring. The metrics for the UK Faculty network are also presented in Fig 9. In this instance, a total of 400 rewirings were conducted.

The analysis underscores the diverse impacts on network metrics, such as assortativity, average closeness, and average local clustering brought by the two randomization alternatives. Random rewiring prompt discernible shifts in metrics, with some experiencing reduction and others augmentation.

Metrics like average betweenness and local clustering show declines due to the loss of central nodes, consequently affecting path lengths and triangle counts. This loss instigates notable alterations in network cohesion and clustering coefficients, observed consistently in randomly rewired networks. Negative assortativity experiences a decline, indicating an inclination toward increased heterogeneity.

These fluctuations can disrupt information flow or community structures within the network, potentially impacting resilience or functional segregation. However, the abruptness of these changes impedes a comprehensive understanding of their implications.

One primary disadvantage of random rewiring is its potential to induce abrupt and less controlled alterations in network topology. While it provides a means to explore variations in network structure, this alternative lacks precision in targeting specific areas or nodes within the network for modification. As a result, the insights derived from random rewiring might not offer a comprehensive understanding of how particular changes impact the network’s behavior or functionality in a targeted manner.

In contrast, the controlled rewiring presents gradual insights into metrics’ average alterations. It primarily targets peripheral edges before affecting core ones, systematically influencing metrics’ average. This alternative surpasses random rewiring by inducing sustained alterations in various metrics, notably in central nodes.

This way, controlled rewiring prevents false positives, as shown in Fig 7, and offers a finer granularity in comprehending network changes, exceeding the effects observed through random alternatives. Its efficacy lies in capturing subtle network modifications, particularly in central nodes, resulting in non-significant deviations from the original network’s structure.

## Discussion

As a result of the conducted experiments, our reference model analysis demonstrates that the combination of the Expanded Quadratic Assignment Procedure (EQAP) controlled rewiring method does not erroneously detect statistically significant relationships within random networks. The EQAP is a novel method for comparing networks, supported by the use of controlled rewiring to ensure the validity of the statistical tests. While controlled rewiring is indeed crucial for generating networks that are structurally similar to the original, it is not merely an illustration of EQAP’s usefulness. Instead, controlled rewiring represents a novel randomization alternative that we need to incorporate alongside EQAP to achieve robust network comparisons. This result validates the effectiveness of the proposed method in preventing false positives or Type I errors, such as incorrectly inferring network effects where none exist (e.g., mistakenly attributing an outcome to network topology) [17, 18]. In contrast to traditional permutation methods, which often yield incorrect conclusions when comparing a network to a version of itself, the methods outlined in this article are gradual. They offer reference models that alter the original network without causing significant changes. This distinction enhances the reliability of our approach.

The combination between EQAP and controlled rewiring, which can be understood as a modified version of the configuration mode, emerges as a promising standard for evaluating the statistical significance of complex real-world networks due to its accurate approach. One of its primary strengths lies in its capacity to disentangle the contributions of individual characteristics and network connections in shaping model outcomes. Delving into whether observed outputs emanate from individual traits or the intricate web of connections among nodes offers valuable insights into the true influencers driving network dynamics and outcomes. The textitasis on highly connected nodes’ significant impact on outcomes underscores the critical role played by network structure in shaping conclusions. By recognizing the weight of these influential nodes, the provided method prompts researchers to delve deeper into understanding network structures and their implications for accurately interpreting outcomes. Moreover, controlled rewiring’s ability to yield stable and convergent results within a few iterations signifies its efficiency and reliability, offering researchers dependable and consistent results without requiring extensive computational resources.

Furthermore, our approach advocates for gradual network transformations (reference models) to monitor metric changes, textitasizing resampling strategies that capture intricate network complexities beyond tabular data. Ultimately, the metrics used in our study offer a more refined tool for capturing complex relationships than conventional statistical measures. Due to this, it offers a comprehensive approach to studying network dynamics, allowing researchers to investigate both the significance of dyadic relationships and the effects of structural changes on network properties. There are different types of hypotheses that the combination of QAP and controlled rewiring can address:

- **Impact of structural changes on dyadic relationships**: Researchers can use EQAP to assess the similarity or dissimilarity between networks before and after controlled rewiring. This allows them to test hypotheses about how specific alterations in network topology influence the strength and significance of dyadic relationships between the two networks [63, 64].
- **Identification of critical edges**: Using EQAP, researchers can identify critical edges and nodes significantly affecting network structure and dynamics. Hypotheses related to the importance of specific edges or nodes in facilitating communication or flow within the network can be tested by systematically rewiring edges and observing changes in network properties [65, 66].
- **Network resilience and robustness**: The integrated methodology can assess hypotheses related to network resilience and robustness to perturbations. Researchers can investigate how different controlled rewiring strategies impact the network’s ability to resist disruptions or maintain functionality [21, 67].
- **Emergence of structural patterns**: Researchers can explore the emergence of structural patterns or configurations by applying EQAP. Hypotheses about the formation of clusters, communities, or motifs in response to specific changes in network topology can be tested by analyzing similarities or dissimilarities between networks [68, 69].
- **Optimization of network structure**: The EQAP enables researchers to test hypotheses related to network optimization and efficiency. By systematically rewiring edges to optimize specific network properties, researchers can assess how changes in network topology affect the strength and significance of dyadic relationships and overall network performance [70, 71].

However, we must acknowledge some caution regarding edge betweenness, which is a valuable centrality measure in network analysis, but its interpretation in directed graphs is complex and context-dependent. In undirected graphs, edge betweenness straightforwardly indicates critical paths for connectivity. However, in directed graphs, the measure’s meaning varies with the nature of the relationships, such as resource flows or transactions. High betweenness in resource flow networks might highlight bottlenecks, while in transactional networks, it could signify key transactional pathways. Analysts must consider these relational contexts to avoid misinterpretation and draw accurate conclusions about network vulnerabilities and optimization opportunities.

## Conclusions

This article textitasizes the crucial role of network structure in influencing processes within it, noting the pitfalls of overlooking its impact on drawing accurate conclusions about causes and consequences. Using a user-friendly Python library, the article introduces the Expanded Quadratic Assignment Procedure (EQAP), a novel statistical tool designed for precise test calculation and interpretation. Illustrated through real-world examples from organizational and social networks, the methodology demonstrates efficacy in analyzing complex networks, ensuring researchers protect against Type I errors when exploring intricate network metrics reliant on topology complexities, such as centrality or clustering coefficients. Although the proposed method is valid indistinctively for directed and undirected networks, further statistical developments are needed. Future steps involve extending the application of our approach to various network types, including weighted, temporal, or multiplex networks. Additionally, we want to incorporate percolation model simulations into the generation of synthetic networks to ensure their structural similarity to the original network.

## Supporting information

S1 File (ZIP)
