# Supplementary material for: Structure matters: Assessing the statistical significance of network topologies
Source: PLoS One. 2024 Oct 2;19(10):e0309005. doi: 10.1371/journal.pone.0309005 (PMC11446434; doi:10.1371/journal.pone.0309005)
Supplement: S1 File — (ZIP) [file pone.0309005.s001.zip › Supporting information.pdf]

Supporting information

S1 Datasets. Enron-Email and UK Faculty friendship datasets. The datasets analyzed during the current study are available in the igraphdata R package.

S2 Python Library. Expanded Mantel test and QAP Repository. We also uploaded the developed Python library to the public Expanded Mantel test and QAP Repository for research reproducibility.

S3 Appendix. The Expanded Mantel test. The Expanded Mantel test loops s times to compute the Mantel correlation coefficients R, its corresponding p-value p, and the new values for the selected metric. To this aim, it takes the distance matrix W from the adjacency matrix A of the network G, a method Δ for changing the matrix, the number of changes n, i.e., the number of rewirings or shuffles, and a topological metric $\mathcal{M}$ as inputs. The algorithm starts by initializing the selected permutation method Δ with n changes. The adjacency matrix A values are randomly changed in each iteration based on the specified permutation method Δ: shuffle, random, or controlled rewiring. The selected $\mathcal{M}$ is recalculated and stored. The changed Mantel correlation coefficient r' is calculated. If r' is greater than or equal to the initial r, the *counter* is incremented by 1. After completing all the simulations, the p-value is calculated as the *counter* to n ratio. Finally, the algorithm returns the Mantel correlation coefficients R, the p-value p, and the recalculated metric measures M. Since the initial r will always be 1 when we compare a network to a version of itself, r' will always be 0, as shown in the limitations section. We also expanded the Mantel test for the completeness of the results.

Algorithm 1 Expanded Mantel test

Require: Distance matrix A, modification algorithm Δ, number of changes n, topological metric $\mathcal{M}$, number of simulations s
Ensure: Recalculated metrics M, Mantel correlation coefficients R, p-value p

```
W ← dist(A, A)                          ▷ Compute the distance matrix
r ← RV(W, W)                            ▷ Compute the initial squared Pearson correlation coefficient
counter ← 0                             ▷ Initialize counter
M, R ← [ ], [ ]                         ▷ Create empty lists
for k ← 1 to s do                       ▷ Number of simulations
    A' ← Δ(A, n)                        ▷ Apply the number of changes
    W' ← dist(A', A')                   ▷ Compute the distance matrix
    M.add(ℳ(W'))                        ▷ Add the recalculated topological metric
    r' ← RV(W, W')                      ▷ Compute the resulting correlation coefficient with the modified matrix
    R.add(r')                           ▷ Add the recalculated correlation coefficient
    if r ≤ r' then                      ▷ Consider only simulations with a resulting correlation coefficient greater than r
        counter ← counter + 1
    end if
end for
p ← counter / s                         ▷ Calculate the p-value
return M, R, p
```
